# Abnormalities of pubertal development and gonadal function in Noonan syndrome

**DOI:** 10.3389/fendo.2023.1213098

**Published:** 2023-07-28

**Authors:** Giuseppa Patti, Marco Scaglione, Nadia Gabriella Maiorano, Giulia Rosti, Maria Teresa Divizia, Tiziana Camia, Elena Lucia De Rose, Alice Zucconi, Emilio Casalini, Flavia Napoli, Natascia Di Iorgi, Mohamad Maghnie

**Affiliations:** ^1^Department of Pediatrics, IRCCS Istituto Giannina Gaslini, Genova, Italy; ^2^Department of Neuroscience, Rehabilitation, Ophthalmology, Genetics, Maternal and Child Health, University of Genova, Genova, Italy; ^3^Department of Clinical Genetics and Genomics, IRCCS Istituto Giannina Gaslini, Genova, Italy

**Keywords:** Noonan syndrome, puberty, gonadal function, vertical transmission, familial cases, genetic counselling

## Abstract

**Background:**

Noonan syndrome (NS) is a genetic multisystem disorder characterised by variable clinical manifestations including dysmorphic facial features, short stature, congenital heart disease, renal anomalies, lymphatic malformations, chest deformities, cryptorchidism in males.

**Methods:**

In this narrative review, we summarized the available data on puberty and gonadal function in NS subjects and the role of the RAS/mitogen-activated protein kinase (MAPK) signalling pathway in fertility. In addition, we have reported our personal experience on pubertal development and vertical transmission in NS.

**Conclusions:**

According to the literature and to our experience, NS patients seem to have a delay in puberty onset compared to the physiological timing reported in healthy children. Males with NS seem to be at risk of gonadal dysfunction secondary not only to cryptorchidism but also to other underlying developmental factors including the MAP/MAPK pathway and genetics. Long-term data on a large cohort of males and females with NS are needed to better understand the impact of delayed puberty on adult height, metabolic profile and well-being. The role of genetic counselling and fertility related-issues is crucial.

## Introduction

Noonan syndrome (NS) is an autosomal-dominant inherited disorder affecting 1:1000 to 1:2500 live births. The clinical phenotype is variable and involves multiple organ systems (1–4). NS belongs to a group of phenotypically overlapping genetic conditions caused by germline mutations in genes encoding components or regulators of the RAS/mitogen-activated protein kinase (MAPK) signalling pathway, collectively known as RASopathies (1–4).

The three most common reported features in this condition are unusual facial features, short stature and congenital heart defects but several other organs may be involved. Since no consensus clinical diagnostic criteria for Noonan syndrome have been published, although scoring systems (5) and UK guidelines are available (6), we aimed to evaluate the clinical context and the degree and extent of gonadal dysfunction in patients with NS.

## Clinical manifestations and genotype

Clinical manifestations vary with the age and include dysmorphic facial features (curly/wooly hair, wide forehead, thickly hooded/prominent eyes, neck skin webbing, small chin), short stature, and congenital heart disease (especially pulmonary valve stenosis, hypertrophic cardiomyopathy); chest deformity (pectus carinatum/excavatum, broad thorax), variable degrees of developmental delay and other features include neonatal feeding difficulties/failure to thrive, cryptorchidism in boys, lymphatic dysplasia and coagulation defects (1–3). The syndrome is typically caused by gain-of-function (activating) mutations in multiple genes in the RAS/MAPK signal transduction pathway.

Noonan Syndrome is caused by an heterozygous pathogenic (or likely pathogenic) variant in one of the associated genes. Genes implicated include: *PTPN11* (accounting for 40-50% of total NS patients) (1–4), *SOS1* (10-20%), *RAF1* (3-17%), *RIT1* (9%), and a group of genes, *SHOC2*, *RASA2, LZTR1, SPRED2, RIT1, SOS2, CBL, KRAS, NRAS, MRAS, PRAS, BRAF, PPP1CB, A2ML1, MAP2K1* and *CDC42* (each of these genes accounting for 1-5% of NS cases or less) (1–4, 7). Most frequently, the causative variant is a single nucleotide variant (SNV) revealed through sequence analysis, although, a few NS and NS–like cases have been reported to show copy number variations (8, 9). In approximately 20–30% of patients with NS, the genetic cause of the disease remains unknown. Noonan Syndrome shows mainly an autosomal dominant inheritance. NS is often sporadic, however 30%-75% of individuals with the condition have an affected parent Due to the high recurrence risk (50% at each pregnancy), it is mandatory to test both parents. More rarely, NS shows an autosomal recessive or digenic inheritance (10), where the parents are usually healthy carriers, with a recurrence risk of 25%. Distinctive features of the different genotypes conditions have been reported showing some phenotype-genotype correlations (11).

## Characteristics of puberty in Noonan syndrome

Children with NS often are referred to the pediatric endocrinologist because of the short stature, undescended testes in males or delayed puberty both in males and females (3). Although height and weight are usually normal at birth, height SD score decreases within the first few months. In general, there is a delay of at least a 2-years between bone age and chronological age, and continued growth may occur into the early 20s (3).

Rezende et al. evaluated 133 Brazilian subjects with a molecular diagnosis of NS and mean age at onset of puberty of 11.9 ± 1.9 years in girls and 12.5± 1.7 years in boys, which was significantly later than the local population (p=0.025, p< 0.001). Girls presented with delayed puberty more frequently than boys and height gain from the onset of puberty to adult height was lower in children with pubertal delay (12). In a study including 65 NS subjects (clinical diagnosis without genetic confirmation), Romano et al. showed that the mean age at Tanner stage II was 13.4 years (range 10.8-16.4 years) in males and 13 years in females (range 10.9-15 years); 35% of males started puberty later than 13.5 years, and 44% of females started puberty later than 13 years. Duration of puberty highly correlated with pubertal height gain in males and females (13).

In a retrospective, multicentre, cohort study including 228 Italian NS subjects with genetic diagnosis of the disease, Libraro et al. showed that at the onset of puberty, NS females had a mean age of 12.1 ± 2.3 years, with a mean bone age (BA) of 11.3 ± 1.7 years, while male patients had a mean age of 12.1 ± 1.3 years, with a mean BA of 10.8 ± 1.3 years. The frequency of delayed puberty was reported in 45% of females and 10% of males (14). In addition, in a group of 12 genetically confirmed NS subjects who reached final height, Tamburrino et al. showed a delay in onset by about 6 months in NS subjects compared to the general population and that pubertal growth had a lowered peak, in particular in males (15, 16).

In a prospective observational study of a large cohort of 97 NS followed for a mean of 12 years, Shaw AC et al. showed that hormone injections had been given to 6 of 97 (6%) individuals to induce pubertal development. In those not receiving exogenous sex hormones, puberty started at a mean age of 14.5 years in males and 14 years in females with a range of 10–18 years in both sexes. It should be considered that only 35% of patients had a genetically confirmed disease (PTPN11) (17). All these data highlight that puberty can be delayed in many patients with NS regardless of gender, and that final height could be compromised due to inadequate height gain at puberty. There are no data to suggest whether cryptorchidism in males was associated with absent or insufficient “Mini puberty”. Clinical and laboratory data suggest that postnatal growth impairment as well as inadequate height gain during puberty can also be explained by a reduction in the IGF1 generation stimulated by GH. Available evidence suggests a partial insensitivity to GH by a post-receptor mechanism in NS (18).

## Gonadal function in Noonan syndrome

There are only scant published data on gonadal and reproductive function and fertility in Noonan syndrome (19, 20).

Ankarberg-Lindgren et al. followed longitudinally 12 NS males from pre-puberty until adulthood showing that at a mean age of 22.3 ± 3.6 years, adult Noonan males showed higher *Luteinizing hormone* (LH), *Follicle stimulating hormone* (FSH), testosterone and estradiol levels, and lower anti-müllerian hormone (AMH) and inhibin B levels than the reference population, indicative of hypergonadotropic hypogonadism. Sertoli cell dysfunction in combination with impaired Leydig cell function reported by the authors in NS group regardless of the presence or absence of cryptorchidism suggested that cryptorchidism was not the only contributing factor for impaired testicular function in males with NS. The limitation of this study is that only the *PTPN11* gene was tested. 5 out of 12 boys had a mutation in this gene and there was no association between the reproductive hormonal profile and *PTPN11* gene mutations (21).

Moniez et al. evaluated the gonadal function of 37 NS males genetically confirmed [*PTPN11* gain of function mutation in 26 (70.3%) patients, *PTPN11* loss of function mutations in 2 (5.4%), *SOS1* mutations in 6 (16.2%), *RAF1* mutations in 2 (5.4%), and *KRAS* mutations in 1 (2.7%)] of whom 16 (43%) had entered puberty at a median age of 13.5 years (range: 11.4–15.0 years). In particular, age at pubertal onset was negatively correlated with BMI SDS (R= −0.541; P = 0.022) and testosterone levels were normal suggesting that Leydig cell function is not affected. In contrast, NS patients had significant lower levels of AMH (mean SDS: −0.6 ± 1.1; P = 0.003) and inhibin B (mean SDS: −1.1 ± 1.2; P < 0.001) compared with the general population, suggesting a Sertoli cell dysfunction. Lower AMH and inhibin B levels were found in NS-PTPN11 patients, whereas these markers did not differ from healthy children in SOS1 patients. No difference was found between cryptorchid and non-cryptorchid patients for AMH and inhibin B levels (p = 0.43 and 0.62 respectively) while 4 PTPN11 patients displayed hypergonadotropic hypogonadism with azoospermia/cryptozoospermia (22). There are two limitations of this study: 1) Except for the NS-PTPN11 patients, the study of genotype–phenotype correlations was limited by the small amount of data in the other subcohorts 2) Several kits were used for hormone assays (retrospective study) (22).

Marcus et al. evaluated the male gonadal function in 9 males with NS (7 *PTPN11*, 1 *BRAF*, 1 not genetically confirmed) aged between13 and 19 years. LH levels were normal in all patients while Inhibin B was low in six males and above the lower limit of normal in two. All 3 men with normal testicular descent (1 *PTPN11*, 1 *BRAF*, 1 not genetically confirmed) showed Sertoli cells dysfunction (raised FSH level in combination with low inhibin β concentration) suggesting that bilateral cryptorchidism is not the only cause of the impairment of testicular function. Since no data regarding semen quality or paternity data are available, these findings showed no direct proof of impaired fertility but are expression of a likely gonadal dysfunction (23).

In the study of Shaw AC et al. including NS 97 patients, of the 18 individuals who had attempt to have children, 12 (67%) had experienced no problems, 2 (11%) had one miscarriage, one had a history of recurrent miscarriages, one pregnancy ended in stillbirth and one woman had difficulty conceiving. One male had a low sperm count, and considering the small number of subjects who wanted to start a family, the impact of cryptorchidism on reproductive function could not be assessed. The major limitation of this study is that that only 35% of this cohort had a genetically confirmed disease (PTPN11) and the number of adults who attempted to have children with genetic confirmation of the disease was not reported (17).

Elsawi et al. in 1994 evaluated genital tract function in 11 men with NS without genetic confirmation of the disease. Testicular maldescent was reported in six of them and puberty was delayed in three (one with history of cryptorchidism). The mean testis volume was 21 ml (range 15-25) and none of the patients reported sexual dysfunction. LH and testosterone levels were normal in all patients, but the FSH levels were raised in all men with cryptorchidism with the exception of one man who had normal sized testes. Four of the men had married, and had fathered children and NS occurred in their offspring confirming paternity. Only one of these fathers belongs to the group of patients with history of cryptorchidism while the other four with history of cryptorchidism had azoospermia (3 patients) or severe oligozoospermia (1 patient), with raised plasma levels of FSH. In this study two men aged 21 and 22 with a history of delayed puberty were unable to produce a sample of semen at the time of their visit. The limitation of this study is the lack of genetic confirmation of NS (24).

These findings suggest different causes of gonadal dysfunction involving either Sertoli or Leydig cells beside the presence or absence of cryptorchidism (22). Available studies on gonadal function in NS published in the last 15 years are summarized in **Table 1**.

**Table 1:** Available studies on gonadal function in Noonan Syndrome published in the last 15 years.

Authors	Year	Methods and Population	Results	Limitations
Moniez et al. (22)	2018	Retrospective study in 37 males with genetically confirmed NS. Serum sexual hormone levels (LH, FSH, testosterone , AMH, inhibin B were analysed	NS patients had significant lower levels of AMH and inhibin B compared with the general population, suggesting a Sertoli cells dysfunction	Except for the NS-PTPN11 patients, the study of genotype–phenotype correlations was limited by the small available data in the other subcohorts. Several kits were used for hormone assays (retrospective study)
Ankarberg-Lindgreen et al. (21)	2011	12 NS males were longitudinally followed from pre/puberty until adulthood. Testicular volume and sexual hormone concentrations were evaluated	Before puberty, reproductive hormone levels (LH, FSH, testosterone, E, AMH, inhibin B were within the expected range in almost all cases In adulthood, NS males had higher LH, FSH, testosterone and E levels and lower AMH and inhibin B levels than the reference population. This study indicates Sertoli cell dysfunction in combination with impaired Leydig cell function in both NS groups (with and without cryptorchidism)	Only PTPN11 gene was tested. 5 out of 12 boys had a mutation in the PTPN11 gene
Marcus et al. (23)	2008	Evaluation of gonadal function in 9 males with NS (7 PTPN11, 1 BRAF, 1 not genetically confirmed) aged from 13 to 19 years	LH levels were normal in all patients. Inhibin B was low in six males and just above the lower limit of normal in two. All 3 men with normal testicular descent (1 PTPN11, 1 BRAF, 1 not genetically confirmed) showed Sertoli cells dysfunction (raised FSH level in combination with low inhibin β concentration) suggesting that bilateral cryptorchidism is not the only cause of the impairment of testicular function	Small number of patients. No possible genotype-phenotype correlation (7 PTPN11, 1 BRAF, 1 not genetically confirmed)

AMH, anti-müllerian hormone; FSH, Follicle stimulating hormone; LH, Luteinizing hormone; E, estradiol.

## Vertical transmission in Noonan syndrome and role of RAS pathway in fertility

The reproductive system in females with Noonan syndromes appears to be normal although the prevalence of fertile or unfertile women with NS is not well understood. Most familial cases that have been reported have shown an overrepresentation of affected mothers. Although males appear to have reduced fertility, male transmission is well described and not uncommon (16, 17, 25–29).

The male infertility described in Noonan males was initially attributed to the presence of cryptorchidism. The observation of the presence of gonadal function alterations even in patients without a history of cryptorchidism led to the hypothesis that other underlying intrinsic and developmental factors including the MAP/MAPK pathway and genetics could also contribute to the gonadal dysfunction described in males with this syndrome (19, 20)

RAS pathway plays a significant role in normal germ cell development affecting proliferation and migration. Abnormal RAS function may result in abnormal Sertoli and germ cell development with or without abnormal testicular descend and can explain the cases of infertility observed in some NS males (19, 20, 30). Puri et al. described the critical role of PTPN11 for the proliferation, self-renewal and differentiation of spermatogonial stem cells that are necessary to replenish the germ cells that will become sperm. Immunofluorescence studies revealed that PTPN11 is expressed in the nuclei and cytoplasm of Sertoli cells and contributes to spermatogonial stem cells self-renewal (31). Additional information regarding PTPN11 functions in Sertoli cells was generated from a transgenic mouse model in which PTPN11 expression was eliminated specifically in Sertoli cells. This model showed that PTPN11 deficiency caused sterility due to an imbalance in the differentiation and self-renewal of spermatogonial stem cells, a dysfunctional blood-testis-barrier and the lack of sperm production (31, 32). With the limitation of the small number of patients, the difference between genes and Sertoli function (impaired in PTPN11 group and not impaired in SOS1 group) described by Moniez et al. suggests a genotype-phenotype correlation (22).

According to Moniez et al., the differences between genotypes may be explained by variations in the degree of activation of the RAS/extracellular signal-regulated kinase (ERK) signalling pathway or by differential, genotype-dependent, tissue specificity (22). In a study in mice Hasegawa et al. showed that MEK/ERK signaling directly and indirectly contributes to the cyclical self-renewal of spermatogonial stem cells by mediating a signal that promotes their periodical self-renewal and proliferation (33)

Data on duck ovaries showed that epidermal growth factor receptor (EGFR) promotes the proliferation of quail follicular granulosa cells through the MAPK/(ERK) signaling pathway (34).

Fan HY et al. showed that MAPK3/1 (ERK1/2) in Ovarian Granulosa Cells are essential for female fertility. Because the signaling molecules RAS and ERK1/2 are activated by an LH surge in granulosa cells of preovulatory follicles, the authors disrupted ERK1/2 in mouse granulosa cells and provided *in vivo* evidence that these kinases are necessary for LH-induced oocyte resumption of meiosis, ovulation, and luteinization (35). In addition, Idrees et al. showed that PTPN11 (SHP2) is indispensable for growth factors and cytokine signal transduction during bovine oocyte maturation and blastocyst development (36).

## Genetic counselling and surveillance

According to the autosomal dominant inheritance, an affected individual has a 50% chance of transmitting the variant, and thus the condition, to each child, male or female. It is therefore important to perform molecular testing in both parents to define the recurrence risk. If the identified pathogenic variant is not detected on the blood samples of the parents, the recurrence risk is slightly higher than that of the general population, taking into account the possibility of gonadal mosaicism (28).

In cases with autosomal recessive inheritance (37, 38), parents of an affected individual are usually carriers of an heterozygous variant and they have, at every pregnancy, a 25% chance of having an affected child, a 50% chance of having carriers and a 25% of having an healthy non carrier child. Individuals with an heterozygous variant can be asymptomatic or can present with mild features (28). The children of an affected individual are obligate heterozygous, and their affected state depends on the other parent.

In case of pregnancy of an affected individual or an at risk relative, genetic counselling is recommended to indicate the available options to monitor the pregnancy. When the causative variant (or variants) is known, invasive prenatal testing on chorionic villus sampling (CVS) or amniotic fluid can determine whether the foetus carries the familial variant and therefore if it is affected. New evidences are emerging on the possible employment of non‐invasive prenatal screening test (NIPT) for single‐gene disorders. Mohan et al. demonstrated the potential value of NIPT in the early detection of NS, as well as other single‐gene disorders (39). When the molecular cause is unknown, high-resolution ultrasound scans are indicated. Ultrasound finding are nonspecific and may include increased nuchal translucency (NT), cystic hygroma, fetal edema, polyhydramnios, hydronephrosis and cardiac anomalies (40).

Early diagnosis, either by invasive or non-invasive genetic testing or by ultrasound scans, leaves the couple with the decision on whether or not to carry on an affected pregnancy. To overcome this decision, preimplantation genetic testing (PGT) can be offered to couples with an high recurrence risk. This option, recently introduced in assisted reproductive technology, is available only when the causative variant is known. PGT is performed on embryo biopsy and afterwards, only non-affected embryos are transferred to the uterus (28, 41).

Recommended surveillance for affected individuals includes auxological, cardiovascular, cognitive, eyes/hearing, orthopedic, coagulation/bleeding evaluations (28, 42).

Bleeding disorders described in NS include thrombocytopenia, platelet dysfunction, von Willebrand disease, factor deficiencies (XI, XII, VIII, V, IX, II, XIII) (43).

In addition, hematologic disorders and increased risk of malignancies are described in NS. The most common hematologic disorders are transient myeloproliferative disorders in neonates and infants, that in 10% of cases can progress to Juvenile Myelomonocytic leukemia (JMML) (26, 42). Other malignancies include acute lymphoblastic and myeloid leukemia, rhabdomyosarcoma (including embryonal rhabdomyosarcoma), neuroblastoma and brain tumors (low-grade gliomas and glioneuronal tumors) (28, 44–48).

According to Kratz et al, NS children are at an eightfold greater risk of developing childhood cancer compared to the general population (48). Because the cancer risk is below 5%, cancer surveillance is not recommended. However, for children with *PTPN11* or *KRAS* pathogenic variants, known for their association with JMML and myeloproliferative disorders, physical exam including the assessment of spleen size and complete blood count every 3-6 months until the age of 5 years is suggested (28, 49).

Sporadic cases of leukemia and solid tumors in the absence of any clinical signs of NS may harbor somatic variants in *PTPN11, KRAS, LZTR1, MRAS, NRAS, BRAF, MAP2K1* (28, 50–52).

In particular, an association between *PTPN11* and acute lymphoblastic and myeloid leukemia as well as an association between *MAP2K1* and ovarian and lung cancers have been described (48–50). Since these somatic variants are not present in the germline, the tumor predisposition is not heritable (28, 50–52).

### Personal experience on pubertal development in Noonan syndrome

We collected retrospective pubertal data in a cohort of 16 subjects with NS who have started puberty (8 females, 8 males). The mean age at the last evaluation was 17.25 ± 4.5 years (minimum 11.5 -maximum 29.8 years). The diagnosis was genetically confirmed in all patients (7 PTPN11, 4 KRAS, 2 BRAF, 2 SHOC2, 1 SOS1). Clinical pubertal onset was defined as testicular volume ≥ 4 ml in boys and breast development (B2 Tanner stage) in females (53, 54). Hormonal parameters including serum LH, 17-β-estradiol, and testosterone were evaluated by chemiluminescent assay (Roche). LH, 17-β-estradiol and testosterone were considered detectable if ≥0.1 U/L, ≥ 5 pg/ml, ≥5 ng/dl, respectively. Bone age was assessed by Greulich and Pyle method (55).

Mean age at puberty onset in the female group was 12.23 years ± 2.7 SD (minimun 8.9-maximum 16), mean age at pubarche was 12.9 years ± 1.8 SD, mean age at dosable (≥0.1 U/L) LH was 11.2 ± 2.4 years, age at dosable 17-β-estradiol (≥ 5 pg/ml) was 12.4± 3.2 years, age at menarche was 16.09± 2.2 years (minimun 12.5- maximum 18 years). Puberty was pharmacologically induced in a girl with panhypopituitarism caused by suprasellar germinoma (case 9, **Table 2**).

**Table 2:** Data of our 16 patients with Noonan Syndrome.

Case	Gene	Mutation	Gender	Age (y*)	Pubertyonset (y*)	Dosable LH (y*)	Dosable E or T (y*)	Age at final height (y)	Pubertal stage at last examination	Last examinationheight (SD)	Δ Target (SD)	Menarche (y)	Cryptorchidism	VT at last examination (ml)
1	SOS1	c.322G>A, p.Glu108Lys	F	20,4	14,6	14,7	14,1	18,2	5	-0,3	0,9	17,0		
2	KRAS	c.436G>T, p.Ala146Ser	F	17,6	9,9	9,9	9,9	16,2	5	-3,4	-2,1	13		
3	BRAF	c.770A>G, p.Gln257Arg	F	16,1	10,5	10,1	10,1	15,4	4	-3,4	-2,8			
4	SHOC2	c.4A>G, p.Ser2Gly	M	23,5	14,2	14,2	14,2	19,6	5	-3,7	-2,5		No	20
5	KRAS	c.101C>G, p.Pro34Arg	F	11,5	8,9	8,4	9,5		2	-4	-4			
6	PTPN11	c.923A>G, p.Ans308Ser	M	14,4	12,1	11,7	11,7		2	-0,7	-0,4		Yes	5
7	PTPN11	c.1403C>T, p.Thr468Met	M	17,5	14,3	14,3	14,3		4	-1,5	-0,1		No	12
8	PTPN11	c.236A>G, p.Gln79Arg	F	15	10,5	9,2	11,5	16,6	5	-1,8	-2,1	12,5		
9	BRAF	c.681G>C, p.Val276Leu	F	20,1	16,0	13,5	18,5		4	-0,6	0,4	18		
10	PTPN11	c.228 G>C, p.Glu76Asp	M	21,9	12,6	10,5	12,6	20,5	5	-2,2	-0,1		No	25
11	PTPN11	c.922A>G, p.Asn308Asp	M	20,0	13,3	13,4	13,4	17,3	5	-2,4	-1		Yes	20
12	PTPN11	c.236 A>G, p.Gln79Arg	F	17,7	14,1	12,7	13,1		4	-1	-1,3			
13	SHOC2	c.4A>G, p.Ser2Gly	M	15,2	14,1	14,2	14,2		3	-3,2	-2,8		No	12
14	PTPN11	c.188A>G, p.Tyr63Cys	M	13,0	12,9	12,9	12,9		2	-1,5	-0,8		No	8
15	KRAS	c.40 G>A, p.Val14Ile	M	14,5	12,7	8,5	12,0		2	-2,3	-3,9		No	6
16	KRAS	c.40G>A p.Val14lle	F	29,8	13,8			17	5	-2,8		16		

M, male; F, female; FSH, Follicle stimulating hormone; LH, Luteinizing hormone; E, estradiol; T, testosterone; VT, testis volume; y*, age in years; SD, standard deviation.

Mean age at puberty onset in males was 13.3± 0.8 years (range 12.1-14.3 years), mean age at pubarche was 14± 1.4 years (range 12-15 years), mean age at dosable LH (≥0.1 U/L) was 12.4 ± 2 years (range 8.5-14.3 years), mean age at dosable testosterone (≥ 5 ng/ml) was 13.1 ± 1 years (range 11.7-14.3 years). 2 out of 8 males have a history of cryptorchidism. The three men that have reached the final height (1 out of 3 with history of cryptorchidism) had a normal value of testosterone (range 577-1045 ng/dl) and normal testicular volume (range 20-25 ml).

The gap between bone age and chronological age was negative at the age of 8–10 years (mean −30±11.6 months; minimum −38 months, maximum −9 months) as well as at the age of 10–14 years (mean −12.4±11.4 months, minimum −28 months, maximum +6 months).

Final height was reached at a mean age of 16.7 ± 1 years in females (minimum 15.4 years, maximum 18.2 years, 5 subjects) and a mean age of 19.1 ± 1.6 years in males (minimum 17.3 years, maximum 20.5 years, 3 subjects), (**Table 2**).

Genetic variant classification in our patients is reported in **Table 3** (56).

**Table 3:** Genetic variant classification in our patients.

Case	Gene	Mutation	classification (ACMG criteria)
1	SOS1	c.322G>A, p.Glu108Lys	Pathogenic (PS2, PS3, PS4, PM2, PM5, PP3, PP4, PP5)
2	KRAS	c.436G>T, p.Ala146Ser	Pathogenic (PM1, PM2, PM5, PP2, PP3, PP4, PP5)
3	BRAF	c.770A>G, p.Gln257Arg	Pathogenic (PS2, PS3, PM1, PM2, PM5, PP2, PP3, PP4, PP5)
4	SHOC2	c.4A>G, p.Ser2Gly	Pathogenic (PS2, PS3, PS4, PM2, PP4, PP5)
5	KRAS	c.101C>G, p.Pro34Arg	Pathogenic (PM1, PM2, PM5, PP2, PP3, PP4, PP5)
6	PTPN11	c.923A>G, p.Ans308Ser	Pathogenic (PM1, PM2, PM5, PP2, PP3, PP4, PP5)
7	PTPN11	c.1403C>T, p.Thr468Met	Pathogenic (PS3, PS4, PM2, PM5, PM6, PP1, PP2, PP3, PP4, PP5)
8	PTPN11	c.236A>G, p.Gln79Arg	Pathogenic (PM1, PM2, PM5, PP2, PP3, PP4, PP5)
9	BRAF	c.681G>C, p.Val276Leu	Likely pathogenic (PM2, PP2, PP3, PP4, PP5)
10	PTPN11	c.228 G>C, p.Glu76Asp	Pathogenic (PS1, PM1, PM2, PM5, PP2, PP3, PP4, PP5)
11	PTPN11	c.922A>G, p.Asn308Asp	Pathogenic (PS2, PS3, PS4, PM1, PM2, PM5, PP1, PP2, PP3, PP4, PP5)
12	PTPN11	c.236 A>G, p.Gln79Arg	Pathogenic (PM1, PM2, PM5, PP2, PP3, PP5)
13	SHOC2	c.4A>G, p.Ser2Gly	Pathogenic (PS2, PS3, PS4, PM2, PP5)
14	PTPN11	c.188A>G, p.Tyr63Cys	Pathogenic (PS1, PS3, PS4, PM1, PM2, PP1, PP2, PP3, PP4, PP5)
15	KRAS	c.40 G>A, p.Val14Ile	Pathogenic (PS2, PS3, PS4, PM1, PM2, PP2, PP3, PP4, PP5)
16	KRAS	c.40G>A p.Val14lle	Pathogenic (PS2, PS3, PS4, PM1, PM2, PP2, PP3, PP4, PP5)

Variant classification and interpretation criteria. PS, pathogenic strong, PM, pathogenic moderate; PP= pathogenic supporting.

PS1=Same amino acid change as a previously established pathogenic variant regardless of nucleotide change, PS2=De novo (both maternity and paternity confirmed) in a patient with the disease and no family history, PS3=Well-established in vitro or in vivo functional studies supportive of a damaging effect on the gene or gene product, PS4=The prevalence of the variant in affected individuals is significantly increased compared to the prevalence in controls, PM1=Located in a mutational hot spot and/or critical and well-established functional domain (e.g. active site of an enzyme) without benign variation, PM2=Absent from controls (or at extremely low frequency if recessive) in Exome Sequencing Project, 1000 Genomes or ExAC, PM5=Novel missense change at an amino acid residue where a different missense change determined to be pathogenic has been seen before, PP1=Co-segregation with disease in multiple affected family members in a gene definitively known to cause the disease, PP2=Missense variant in a gene that has a low rate of benign missense variation and where missense variants are a common mechanism of disease, PP3=Multiple lines of computational evidence support a deleterious effect on the gene or gene product (conservation, evolutionary, splicing impact, etc), PP4=Patient’s phenotype or family history is highly specific for a disease with a single genetic etiology, PP5=Reputable source recently reports variant as pathogenic but the evidence is not available to the laboratory to perform an independent evaluation (56).

## Conclusion

According to the literature and to our experience, NS patients seem to have a delay in puberty compared to the physiological timing reported in healthy children (57). Males with NS seem to be at risk of gonadal dysfunction secondary to cryptorchidism or to other underlying developmental factors including the MAP/MAPK pathway and genetics. The latter hypothesis is supported by the observation of the key role of the RAS/MAPK pathway and PTPN11 gene on Sertoli cell development and spermatogonial stem cells self-renewal (20, 30, 31).

Long-term data on a large cohort of males and females with NS are needed to better understand the impact of delayed puberty on adult height, cardio-metabolic risk factors, bone health and well-being. The role of genetic counselling and fertility related-issues remain crucial.

## Data availability statement

The raw data supporting the conclusions of this article will be made available by the authors, without undue reservation.

## Author contributions

GP and MM conceived the idea for the topic, both contributed to the outline; MS, NM, ER, TC, AZ helped to write the first draft, GR and MD helped with the genetic investigations and the drafting of the genetic counselling section, FN and NI contributed to the multiple revisions. All authors contributed to the article and approved the submitted version.
